# Exploring first-feeding diets for European eel larval culture: Insights at morphological, nutritional, and molecular levels

**DOI:** 10.1371/journal.pone.0283680

**Published:** 2023-04-27

**Authors:** Elisa Benini, Kasun A. Bandara, Sebastian Nikitas Politis, Sofia Engrola, Anders Nielsen, Luis E. C. Conceição, Andre Santos, Sune Riis Sørensen, Jonna Tomkiewicz

**Affiliations:** 1 National Institute of Aquatic Resources, Technical University of Denmark, Lyngby, Denmark; 2 Centre of Marine Sciences, Universidade do Algarve, Faro, Portugal; 3 SPAROS Lda, Área Empresarial de Marim, Lote C, Olhão, Portugal; National Cheng Kung University, TAIWAN

## Abstract

Closing the life cycle of European eel (*Anguilla anguilla*) in captivity is targeted to provide a sustainable, year-round supply of juveniles for aquaculture. Present focus is on the nutritional requirements during the larval first-feeding period. In this study, three experimental diets were tested on hatchery-produced European eel larvae from the onset of the first-feeding stage commencing 10 days post hatch (dph) until 28 dph. Larval mortality was recorded daily, while sampling was conducted at regular intervals to record larval biometrics and analyze the expression of genes related to digestion, appetite, feed intake and growth. Two periods of high mortality were identified: the first appeared shortly after introduction of feeds (10–12 dph), while the second occurred 20–24 dph, indicating the “point of no return”. This interpretation was supported at the molecular level by the expression of the gene encoding the “hunger hormone” ghrelin (*ghrl*) that peaked at 22 dph in all dietary trials, suggesting that most larvae were fasting. However, in larvae fed diet 3, *ghrl* expression was downregulated beyond 22 dph, which indicated that those larvae were no longer starving at this stage, while upregulation of genes encoding the major digestive enzymes (*try*, *tgl*, and *amyl2a*) advocated their healthy development. Moreover, for larvae fed diet 3, the expression of those genes as well as genes for feed intake (*pomca*) and growth (*gh*) continued to increase towards 28 dph. These results together with the registered highest survival, largest dry weight increase, and enhanced biometrics (length and body area) pointed to diet 3 as the best-performing. As a whole, this first-feeding study represents a landmark being the first to document European eel larval growth and survival beyond the point of no return, providing novel insights into the molecular development of digestive functions during the first feeding stage.

## Introduction

European eel (*Anguilla anguilla*) is listed as critically endangered and subjected to fisheries and trade restrictions due to a drastic decline of the stock [1, 2]. Therefore, closing the life cycle of European eel in captivity is targeted to promote sustainable aquaculture of this species and essential to resolve current limitations of aquaculture production and revive market potentials [3]. Presently, the development of hatchery technology has incrementally advanced, thereby meeting challenges caused by limited knowledge about the biology of the species and its complex catadromous life cycle [4, 5]. Methodologies using assisted reproduction to obtain viable offspring in captivity have been established for both European and Japanese eel (*A*. *japonica*), thereby overcoming the hormonal inhibition, characterizing the onset of their migratory silvering phase [6]. Still, the culture of offspring, in particular the protracted migratory larval stage, represents a major challenge in culture.

For Japanese eel, the life cycle has been closed in captivity, focusing now on mass production of glass eel [7], while research on European eel targets offspring culture. For the latter species, the embryonic phase lasts 56 hours at a rearing temperature of 18°C before hatching into an undeveloped larva with a prominent yolk sac [8, 9]. The newly hatched yolk-sack larvae develop into the feeding stage after 10 to 12 days [9], where they enter the so-called first-feeding window [10]. Here, they need to successfully initiate feeding in order to develop into the leaf-shaped leptocephalus larvae, characteristic of the Elopomorpha superorder [11]. At present, a main bottleneck for progressing feeding culture of European eel larvae is the identification of a suited diet and the establishment of an adequate feeding regime. An obstacle in this context is the limited knowledge about the natural diet of eel leptocephali, in particular for the first feeding stage, but also during their long-lasting migratory phase from oceanic spawning areas towards continental waters, where they metamorphose into glass eels.

Over time, various studies have aimed at determining the natural diet of anguilid leptocephalus larvae. Initial records showed that larval guts appeared mostly empty or contained an undefined substance [11, 12]. Later, it was proposed that particulate organic matter (POM) was the main source of eel larval nutrition, possibly in the form of marine snow, larvacean houses, and/or zooplankton fecal pellets [13, 14]. This theory has been nourished by recent studies using molecular techniques [15, 16]. However, the knowledge gathered in relation to the natural diet of eel larvae has not found implementation into prototype feeds. In fact, analyses of the bulk nitrogen stable isotopes of the diet used in successful rearing of Japanese eel larvae in captivity revealed that it is far from the anticipated natural feed sources [17].

The diet leading to the first successful culture of Japanese eel larvae, enabling larvae to survive up to 26 days post hatch (dph), was based on egg yolk of spiny dogfish, *Squalus acanthias* [18]. Further modifications of the diet formulation (e.g. addition of soybean peptides, krill extract, and a mixture of vitamins and minerals), resulted in improved growth (22 mm in length) and survival until 100 dph [19]. Subsequent diets, replacing soybean peptides with chicken egg albumin peptides and chitin oligosaccharides, improved the survival and growth of Japanese eel larvae [20], leading to metamorphosis into glass eels around 130 dph. However, using such liquid diets, containing spiny dogfish egg yolk, tend to compromise water quality in the rearing tanks [4]. To ensure adequate water quality, remaining feeds in the tanks need to be flushed away after each repeated daily feeding event and tanks must be replaced daily [7]. Such incremental improvements have enabled captive production of Japanese glass eels, which subsequently led to closing the life cycle in captivity [4].

For the European eel, the development of feeding larval culture is in progress. Thus, recent studies have generated useful insights at the morphological and physiological level. Pioneering studies by [21] and [10] showed that despite larvae successfully ingesting a paste made of rotifers, *Brachionus plicatilis*, they did not survive past 24 dph. Moreover, it was revealed that genes encoding the major digestive enzymes were expressed before the actual initiation of feeding, which indicated that larvae have the potential to process feed items before the end of the yolk sac stage [10]. In this regard, pre-feeding protocols were explored as a strategy to promote feeding initiation and thus, transition towards exogenous feeding [22]. The results of this study suggested that early availability of dietary nutrients can influence the molecular ontogeny of feeding related processes.

In this context, the present study aimed at exploring first-feeding diets and feeding regimes suited for European eel larval culture. Three diets were formulated and tested experimentally (in triplicates), ranging from the onset of the first feeding stage (at 10 dph) until 28 dph. In order to follow larval growth and the ontogeny of key molecular processes and functions, larvae were sampled on day 0, 9, 15, 22 and 28 dph for biometric and gene expression analyses. The combination of genes was selected to target appetite [ghrelin (*ghrl*)], feed intake [proopiomelanocortin (*pomc*)], digestion [trypsin (*try*), triglyceride lipase (*tgl*), amylase (amyl2a)], energy metabolism [cytochrome-c-oxidase (*cox1*)], and growth [growth hormone (*gh*)] related functions and processes.

## Materials and methods

### Ethic statement

Eel in all stages were handled in accordance with the European Union regulations concerning the protection of experimental animals (Dir 2010/63/EU). The experimental protocols were approved by the Animal Experiments Inspectorate of the Danish Ministry of Food, Agriculture and Fisheries (permit number: 2020-15-0201-00768). Eels were anesthetized in relation to tagging, biopsy, and stripping of gametes, and euthanized after stripping (females) or at the end of the experiment (males) by submergence in an aqueous solution of ethyl p-aminobenzoate (benzocaine, 20 mg/L, Sigma Aldrich, Germany). Larvae were anesthetized and euthanized using tricaine methanesulfonate (MS-222, Sigma Aldrich, Germany) at a concentration of 7.5 and 15 mg/L, respectively.

### Broodstock management, gamete production and offspring culture

Female broodstock comprised silver eels caught in Saltbæk Vig, a brackish lake in Zealand, Denmark, through commercial fisheries performed according to the regulations of the Danish Fisheries Act, while male broodstock eels, raised in captivity from the glass eels stage, were obtained from a commercial eel farm (Royal Danish Fish, Hanstholm, Denmark). Female eel were selected based on silvering characteristics, e.g., color and eye size, while male eel were identified based on their smaller size at age (dimorphism) as well as eye and fin characteristics [23, 24]. Once transported to the EEL-HATCH facility (Hirtshals, Denmark), the broodstock was acclimatized to seawater (36 psu) and a temperature of 20°C, before assisted reproduction protocols were applied to initiate gametogenesis. For females, weekly injections of salmon pituitary extract (Argent Aquaculture LLC, Washington, USA) at a dose of 18.75 mg/kg initial body weight were applied to induce vitellogenesis [25], while final maturation was induced using 17a,20b-dihydroxy-4-pregnen-3-one (DHP crystalline, Toronto Research Chemicals, Canada) [26]. For males, weekly injections of human chorionic gonadotropin were applied to induce spermatogenesis (Sigma-Aldrich Chemie, Steinheim, Germany) at 1.5 IU/g initial body weight [27].

Gametes were strip-spawned and fertilized at 20°C using a standardized sperm to egg ratio with gamete contact time of ~5 min [28]. Here, pooled milt from three-five males was added to fertilize the batch of eggs [29]. Thereafter, incubation followed the protocol described by [10], where temperature was lowered to 18°C [9] and light kept below ~10 lx [30]. Hatching occurred at ∼56 hours post fertilization (hpf). Subsequently, the newly hatched larvae were transferred into 77 L tanks, connected to a recirculating aquaculture system (RAS), where temperature was maintained at 18–20°C [9] and salinity at ~36 psu [8]. Water flow was set to 600 mL/min, while light was kept below ~10 lx [30].

### Selection of larvae for the experiment

A batch of high-quality larvae was selected based on hatching success (76%), which was estimated as the percent of embryos successfully hatching between 48 and 60 hpf [31]. Maternal total length and weight were 66 cm and 570 g, respectively, while body length and weight (mean ± SD) of males (*n* = 3) were 42.7 ± 2.1 cm and 136.5 ± 12.23 g, respectively.

### Experimental design and first-feeding regimes

At the end of day 9 post hatch, the larvae were transferred to replicated Kreisel tanks (*n* = 9) at a density of ~60 larvae/L and randomly connected to one of three separate, but identical 0.65 m^3^ RAS units, filled with conditioned filtered seawater [10]. Each RAS unit represented one of three experimental dietary treatments and was connected to three Kreisel tanks (8 L volume). In these systems, salinity was maintained at ~18 psu [20, 32], temperature at ~20°C [9], and light (~500 lx) was only turned-on during feeding [21, 33]. Flow rates were kept at ~ 420 mL/min.

Larval feeds comprised three different liquid diets, pipetted on the bottom of the tank. In each Kreisel tank, food was added at a concentration of 0.5 mL diet per L of water five times per day at two-hour intervals. Here, the light was turned-on and the water current stopped to give larvae the possibility to feed for 30 min. The remaining feed was flushed away using a gentle jet of water. Thereafter, outlets of the Kreisel tanks were disconnected from the corresponding RAS units, so that the rearing water was allowed to run out of the tank for 30 min. Then, seawater pre-adjusted to 20°C and 18 psu was used to refill each RAS unit. Subsequently, the light was turned off and water flow resumed. Larvae were moved into clean tanks daily.

### Composition of diets

All diets were freshly prepared daily. The biochemical composition of each diet is described in Table 1 and the composition of specific fatty acids in Table 2. All diets were adjusted to reach a similar semi-liquid consistency using reverse osmosis water (JG Wasseraufbereitung 600, 2500 L/d, WaterQuality^®^ and RoHS certified, Germany). The composition of diet 1, resembling the recipe for the Japanese eel larval diet [18, 19], contained spiny dogfish eggs (Sterna Seafood AS, Snarøya Norway), krill extract and soybean peptide (99.9% purity, Sgonek Biological Technology Co. Ltd, China). The krill extract consisted of thawed, deskinned krill (Akudim, Esbjerg, Denmark), mixed with reverse osmosis water at a 1:2 ratio, sieved through a sock net (0.2 mm mesh size) and heat-treated for 30 min at ~60°C. Diets 2 and 3 represented modifications of diet 1. Those two diets contained 30% lipid, in accordance with the results obtained by [34] and 60% protein. However, the protein source and molecular size differed between diet 2 and 3. Diet 2 contained fish hydrolysate (CPSP90, Sopropeche, France) encapsulated in whey (Volacactive UltraWhey 80 Instant, Volac International Ltd, Hertfordshire, UK) [35], with molecular weight of approximately 3 kDa whereas Diet 3 contained only whey with a molecular protein weight ranging between 10 and 12 kDa. Whey is a by-product of cheese making or casein in dairy production [36]. The fatty acid composition was relatively similar among diets. Overall, diet 1 contained less protein and more lipids compared to the other diets. Feed formulation details can be provided upon request, for non-commercial purposes.

**Table 1:** Proximal composition of three experimental diets used as feeds for European eel (*Anguilla anguilla*) larvae.

	Diet 1	Diet 2	Diet 3
**Dry mater (%)**	27.1 ± 0.12	29.8 ± 0.09	36.01 ± 0.10
**Protein (%)**	50.89 ± 0.35	61.12 ± 0.17	59.09 ± 0.81
**Lipid (%)**	37.52 ± 0.25	27.49 ± 0.10	27.51 ± 0.28
**Ash (%)**	3.33 ± 0.04	3.21 ± 0.12	2.91 ± 0.03
**Energy (kj/g)**	29.30 ± 0.18	27.93 ± 0.17	28.46 ± 0.18

**Table 2:** Fatty acids of three experimental diets applied as feeds for larvae of European eel, *Anguilla anguilla*.

	Diet 1	Diet 2	Diet 3
**14:0**	1.82	2.65	2.68
**15:0**	0.83	0.85	0.96
**16:0**	85.97	92.51	94.08
**18:0**	24.80	24.95	26.29
**24:0**	5.07	6.27	5.39
**Σ SFA**	118.49	127.25	129.41
**14:1**	0.00	0.00	0.00
**16:1**	11.77	12.73	11.58
**18:1n-9**	114.51	114.81	113.89
**20:1**	42.82	37.11	42.21
**22:1**	13.67	8.31	12.38
**24:1**	4.04	3.95	3.94
**Σ MUFA**	186.82	176.92	184.01
**18:2n-6**	9.00	9.25	8.50
**18:3n-6**	2.30	1.32	1.83
**20:2n-6**	0.00	0.00	0.00
**20:3n-6**	0.00	0.00	0.00
**20:4n-6**	18.53	19.01	18.78
**Σ (n-6) PUFA**	29.83	29.58	29.10
**18:3n-3**	2.35	2.77	1.84
**18:4n-3**	2.15	2.37	2.57
**20:4n-3**	3.19	3.70	3.48
**20:5n-3**	55.10	53.13	63.86
**22:5n-3**	18.89	20.85	19.91
**22:6n-3**	104.76	105.57	101.29
**Σ (n-3) PUFA**	186.44	188.39	192.96
**Σ PUFA**	216.27	217.97	222.06
**Σ FA**	555.59	546.89	565.80

Each class of fatty acids is expressed in relation to the amount of total lipid (mg FA/ mg lipid). SFA: saturated fatty acids; MUFA: monounsaturated fatty acids; PUFA: polyunsaturated fatty acids; FA: fatty acids.

### Larval survival and biometry

From 10 dph and onwards, dead larvae were counted and removed daily from all experimental units (*n = 9*). Larval cumulative mortality was calculated as a percentage from 10 until 28 dph. Larvae sampled throughout the experimental period were not considered for further calculation. Subsamples (*n* = 3) of 10 larvae each were collected at hatch (0 dph) and larvae photographed for subsequent measurement of initial larval biometry. Thereafter, pools of 10 larvae per replicated tank (*n* = 3) and diet (*n* = 3) were sampled and photographed at selected developmental stages, including the beginning of the first-feeding window, but before introduction of the experimental diets (9 dph), the middle of the first-feeding window (15 dph), the end of the first-feeding window (22 dph) and beyond the first-feeding stage (28 dph), passing the point of no return. All images were taken using a digital camera (Digital Sight DS-Fi2, Nikon Corporation, Japan) attached to a zoom stereomicroscope (SMZ1270i, Nikon Corporation, Japan). NIS-Elements D analysis software (Nikon Corporation, Japan, Version 3.2) was used to assess larval biometrics from the images, such as standard length and body area, while feeding incidence and gut fullness at 15 and 22 dph was calculated as described in [21].

### Dry weight

At 9, 15 and 22 dph, 10 larvae per replicated tank (*n* = 3) and per diet (*n* = 3) were euthanized, directly frozen at -80°C and subsequently, freeze-dried using a laboratory freeze-dryer (Christ Beta 2, Martin Christ Gefriertrocknungsanlagen GmbH, Osterode am Harz, Germany). Freeze-dried larvae were weighed using a microbalance (Mettler-Toledo A/S, Denmark). The individual dry weight was calculated by dividing the dry weight of each larval pool by the number of larvae in the sample.

### Gene expression

For molecular analysis, 10–15 larvae per diet were sampled at selected time points (referred to as age), i.e., before introduction of feeding (0 and 9 dph) and throughout the first-feeding window (15 and 22 dph). At 28 dph, there were not enough larvae left in the treatments diet 1 and 2 to sample for molecular analyses. Therefore, gene expression at this time point was conducted only for larvae fed diet 3. Sampled larvae were euthanized using MS-222, rinsed with deionized water, preserved in RNA later (Stabilization Reagent) and kept at -20°C, following the procedure provided by the supplier (Qiagen, Germany). RNA was then extracted using the NucleoSpin RNA Kit (Macherey-Nagel, Germany) following the manufacturer’s instructions. RNA concentration and purity (260/280 = 2.09 ± 0.03, 230/260 = 2.02 ± 0.12) were determined by spectrophotometry using Nanodrop ND-1000 (Peqlab, Germany) and normalized to a common concentration of 100 ng/μl with HPLC water. From the resulting total RNA, 450 ng were transcribed using the qScript^™^ cDNA Synthesis Kit (Quantabio, Germany) according to the manufacturer’s instructions, including an additional gDNA wipeout step prior to transcription [PerfeCtaR DNase I Kit (Quantabio, Germany)].

The expression levels of seven target and three reference (housekeeping) genes were determined by quantitative real-time PCR (qRT-PCR) using specific primers. Primers were designed using Primer3 software v 0.4.01 based on cDNA and predicted cDNA sequences available in Genbank databases (Table 3). All primers were designed for an amplification size ranging from 75 to 200 nucleotides. Generally, mean (± SE) primer efficiency was 93 ± 6%. The elongation factor 1 a (*ef1a*), 40S ribosomal S18 (*rps18)* and tubulin β1 (*tubβ*) genes were chosen as reference genes, as they have been previously suggested to be the most stable in fish larvae and thus, the most reliable reference genes [37]. Their stability of reference genes was statistically tested (one-way ANOVA) and the expression was not significantly different across treatments.

**Table 3:** Sequences of European eel, *Anguilla anguilla* primers used for amplification of genes by qRT-PCR. Primers were designed based on sequences available on Genbank databases.

Full name	Function	Abbreviation	Primer sequence Forward	Primer sequence Reverse	Accession number
**18s ribosomal RNA**	Reference	*rps18*	ACGAGGTTGAGAGAGTGGTG	TCAGCCTCTCCAGATCCTCT	XM_035428800.1
**Elongation factor 1**	Reference	*ef1*	CTGAAGCCTGGTATGGTGGT	CATGGTGCATTTCCACAGAC	XM_035428274.1
**Tubulin β**	Reference	*tubβ*	TGATGACACGGTATTGACC	TGGCACATACTTCCACCG	XM_035419873.1
**Growth hormone**	Growth	*gh*	GTTTGGGACCTCTGATGGGA	AGCAGGCCGTAGTTCTTCAT	XM_035398906.1
**Cytochrome-C-Oxidase**	Energy	*cox1*	CTACTCCTCTCCCTGCCAGT	CTTCTGGGTGGCCGAAGAAT	YP_163818.1
**Prepro-Ghrelin**	Appetite	*ghrl*	TCACCATGACTGAGGAGCTG	TGGGACGCAGGGTTTTATGA	XM_035381207.1
**Proopiomelanocortin**	Feed intake	*pomca*	GCCTGTGCAAGTCTGAACTG	GACACCATAGGGAGCAGGAA	XM_035421304.1
**Amylase**	Digestion	*amyl2a*	AGACCAACAGCGGTGAAATC	TGCACGTTCAAGTCCAAGAG	XM_035420193.1 v3
**Triglyceride lipase**	Digestion	*tgl*	CTGACTGGGACAATGAGCGT	CGTCTCGGTGTCGATGTAGG	XM_035399731.1
**Trypsin**	Digestion	*try*	CTGCTACAAATCCCGTGTGG	GGAGTTGTATTTGGGGTGGC	XM_035429595.1

For larval samples of each tank (*n* = 3), diet (*n* = 3) and age (*n* = 5), expression of genes was analyzed in three technical replicates using the qPCR Biomark^™^ HD system (Fluidigm) based on 96.96 dynamic arrays (GE chips). In brief, a pre-amplification step was performed with a 500 nM primer pool of all primers in TaqMan-PreAmp Master Mix (Applied Biosystems) and 1.3 mL cDNA per sample for 10 min at 95°C; 14 cycles: 15 sec at 95°C and 4 min at 60°C. Obtained PCR products were diluted 1:10 with low EDTA-TE buffer. The pre-amplified product was loaded onto the chip with SSofast—EvaGreen Supermix low Rox (Bio Rad) and DNA-Binding Dye Sample Loading Reagent (Fluidigm). Primers were loaded onto the chip at a concentration of 50 mM. The chip was run according to the Fluidigm 96.96 PCR protocol with a Tm of 60°C. The relative quantity of target gene transcripts was normalized and measured using the 2^-ΔΔCt^ method [38]. Coefficient of variation (CV) of technical replicates was calculated and checked to be < 0.04 [39].

### Statistical analysis

All data were analyzed using R studio statistical analysis software (Version 1.3.959, *RStudio*: *Integrated Development for R*. *RStudio*, *PBC*, *Boston*, *MA*). Residuals were evaluated for normality (Shapiro–Wilk test) and homoscedasticity (plot of residuals vs. predicted values) to ensure they met model assumptions. Data were log (10) transformed to meet these assumptions when necessary. Alpha was set at 0.05 for testing main effects and interactions. Treatment/Diet means were contrasted using Tukey’s Honest Significant Difference test. Standard length, body area, dry weight as well as gene expression (7 genes) at each age (0, 9, 15, 22 and 28 dph) were analyzed using a series of mixed model ANOVAs (PROC GLM). The main model variables were treatment (diet 1 *vs*. diet 2 *vs*. diet 3) and age (0, 9, 15, 22 and 28 dph), while replicated tanks were considered random. The initial model tested included an interaction effect between treatment and age. The model was reduced when possible. The final model was validated through analyses of the residuals. As for survival and daily mortality, the interaction effect between treatments and age was tested and a series of pairwise t-tests were run at all developmental stages (from 10 to 27 dph).

## Results

### Biometry

#### Length

**Fig 1:**
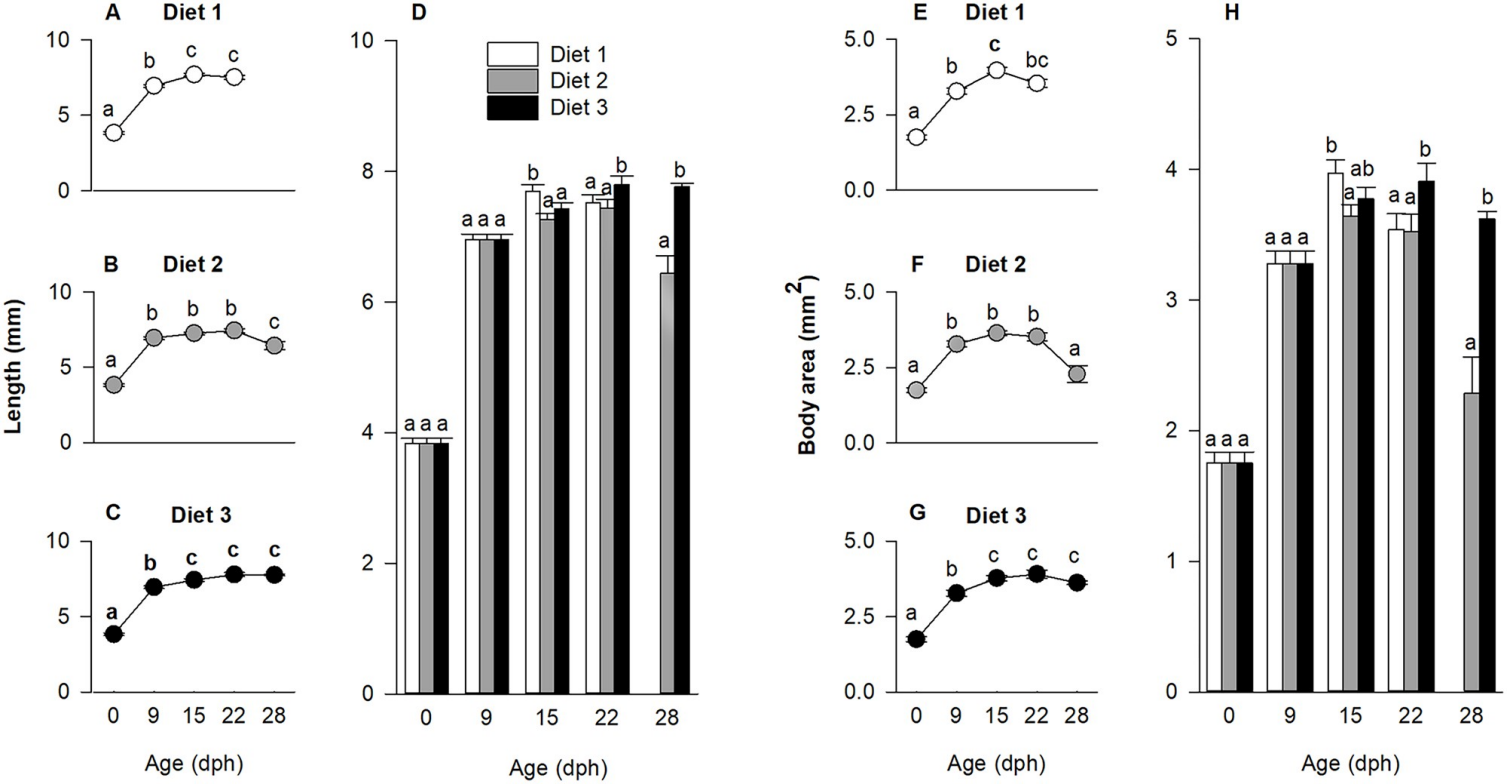
Length and body area in relation to age for larvae of European eel, *Anguilla anguilla*, fed three experimental diets 1, 2 and 3. Effect of age for each diet on standard length (A-C) and body area (E-G) and effect of diets at each age for standard length (D) and body area (H). Assessment occurred at specific developmental points such as hatch (0 days post hatch (dph)), before first-feeding (9 dph), during first-feeding (15 dph), end of the first-feeding period (22 dph) and beyond the first-feeding period (28 dph). Values represent means (± SEM), while different lower-case letters represent significant differences (p < 0.05).

Larval length, measured at hatch (0 dph) and at regular time points throughout the experiment (9, 15, 22 and 28 dph), was influenced by the diet × age interaction (p < 0.01). The model was therefore decomposed into a series of reduced ANOVA models to determine effect of age on length for each diet (Fig 1A–1C) and effect of diets on length at each age (Fig 1D), separately. As marked in (Fig 1A–1C), larval length increased with age from an average of 3.83 ± 0.08 mm at hatch to 6.59 ± 0.09 mm at 9 dph (p < 0.01). During the feeding period, larvae fed diet 1 grew longer between 9 and 15 dph (p < 0.001), while no difference in length was observed between 15 and 22 dph. Subsequently, the larvae died out. For larvae fed diet 2, no increase in length was observed between 9 and 22 dph, while a decrease was detected between 22 and 28 dph (p < 0.01). For larvae fed diet 3, length increased between 9 and 15 dph (p < 0.001), hereafter, remaining stable until the end of the experiment (28 dph). Overall, diets influenced larval length during the feeding period, as shown in Fig 1D, where the longest larvae at 15 dph were the ones fed diet 1 (p < 0.01), while at 22 and 28 dph, the longest larvae were the ones fed diet 3 (p < 0.01).

#### Body area

Body area, measured at hatch (0 dph) and during larval ontogeny (at 9, 15, 22 and 28), was influenced by the diet × age interaction (p < 0.001). Therefore, the model was decomposed into a series of reduced ANOVA models to determine the effect of age on body area for each diet (Fig 1E–1G) and the effect of diets on body area at each age (Fig 1H). As evident in Fig 1E–1G, larval body area increased for all larvae from hatch until 9 dph (p < 0.001). Thereafter, body area of larvae fed diet 1 increased between 9 and 15 dph (p < 0.01) and remained stable until 22 dph. For larvae fed diet 2, similarly, no change in body area was noted between 15 and 22 dph, while a decrease was observed between day 22 and 28 dph (p < 0.01). For larvae fed diet 3, body area increased between 9 and 15 dph (p < 0.01) and remained constant until the end of the experiment (28 dph). Significant differences in body area between dietary treatments were observed at 15 dph, where larvae fed diet 1 had a larger body area compared to diet 2 (p < 0.001). Subsequently, larvae fed diet 3 had larger body area on 22 and 28 dph (p < 0.0001) compared to larvae from the other treatments (Fig 1H).

### Survival

**Fig 2:**
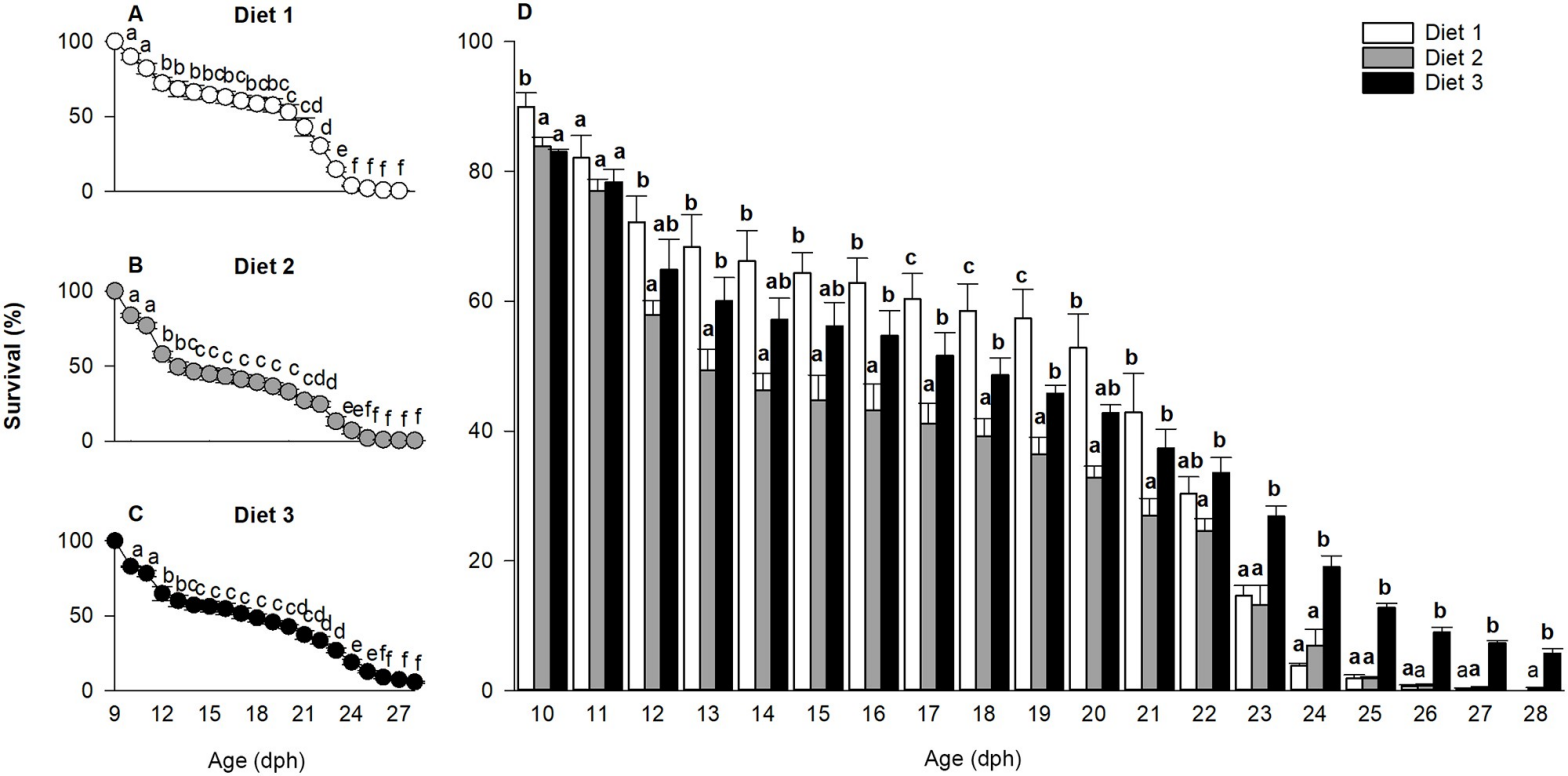
Effect of age for each diet (A, B, C) and effect of diets at each age (D) on European eel, *Anguilla anguilla*, larval survival. Values represent means (± SEM), while different lower-case letters represent significant differences (p < 0.05).

Survival (%) was also significantly affected by the age × treatment interaction (p < 0.001) and thus, a series of pairwise t-tests were run at all developmental stages (Fig 2). Overall, the survival rate significantly decreased with development, however, between 13 and 19 dph, it remained relatively stable within all three dietary regimes (Fig 2A–2C). At 10 dph and between 12 and 16 dph, the survival rate of larvae fed diet 1 was higher than for larvae fed diet 2, but comparable to larvae fed diet 3. However, as evident from Fig 2D, beyond 22 dph, the highest survival rate was observed for larvae fed diet 3 (p < 0.01). At 28 dph, the survival of larvae fed diet 3 and diet 2 was 4% and 0.5%, respectively, while no larvae survived to this age when fed diet 1 (Fig 2D).

### Feeding incidence, gut fullness and growth rate

**Fig 3:**
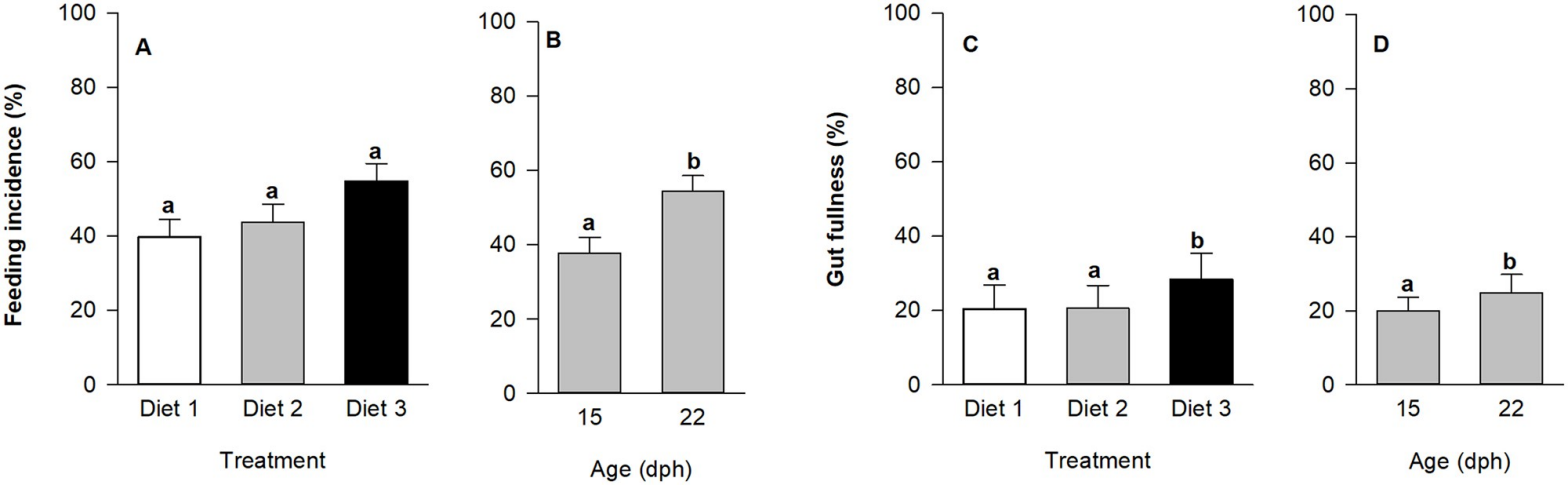
Effect of age and treatment (diet) on larval European eel, *Anguilla anguilla*, feeding incidence (%) and gut fullness (%). Values represent means (± SEM), while different lower-case letters represent significant differences (p < 0.05).

Overall feeding incidence (%) did not differ between dietary treatments (Fig 3A), but increased from 37.8 ± 4.3% to 54.4 ± 4.4% between 15 and 22 dph (Fig 3B; p < 0.001). Moreover, gut fullness (%) was affected by both diet and age, where it increased throughout development (Fig 3D; p < 0.05) and was higher in larvae fed diet 3 compared to larvae fed diet 1 and 2 (Fig 3C; p < 0.02).

### Dry weight

**Fig 4:**
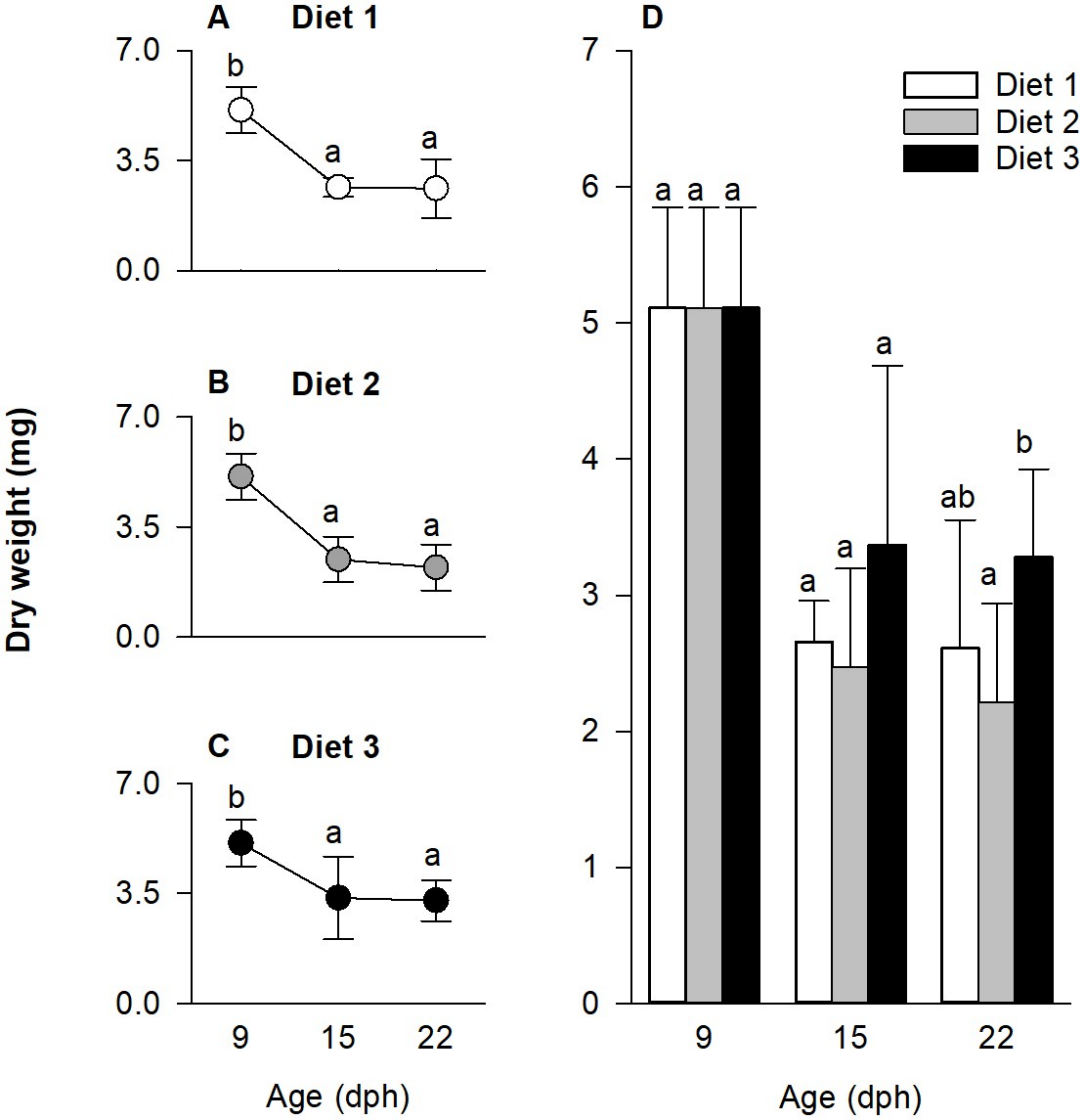
European eel, *Anguilla anguilla*, dry weight (mg/ind) during larval development. Effect of age at each diet (A-C) and effect of diets at each age (D). Values represent means (± SEM), while different lower-case letters represent significant differences (p < 0.05).

Larval dry weight, measured at 9, 15 and 22 dph, was affected by the diet × age interaction (p < 0.001). As such, the model was decomposed to detect the effect of age on dry weight for each diet (Fig 4A–4C) and the effect of diet on dry weight at each age (Fig 4D). As shown in Fig 4A–4C, irrespectively of the diet used, the larval dry weight decreased throughout development (p < 0.01). As evident in Fig 4D, the difference between treatments at 15 dph was not statistically significant, while at 22 dph, the dry weight of larvae fed diet 3 was higher than of larvae fed diet 2, with diet 1 fed larvae being intermediate (p = 0.0087).

### Gene expression

#### Genes related to digestion

**Fig 5:**
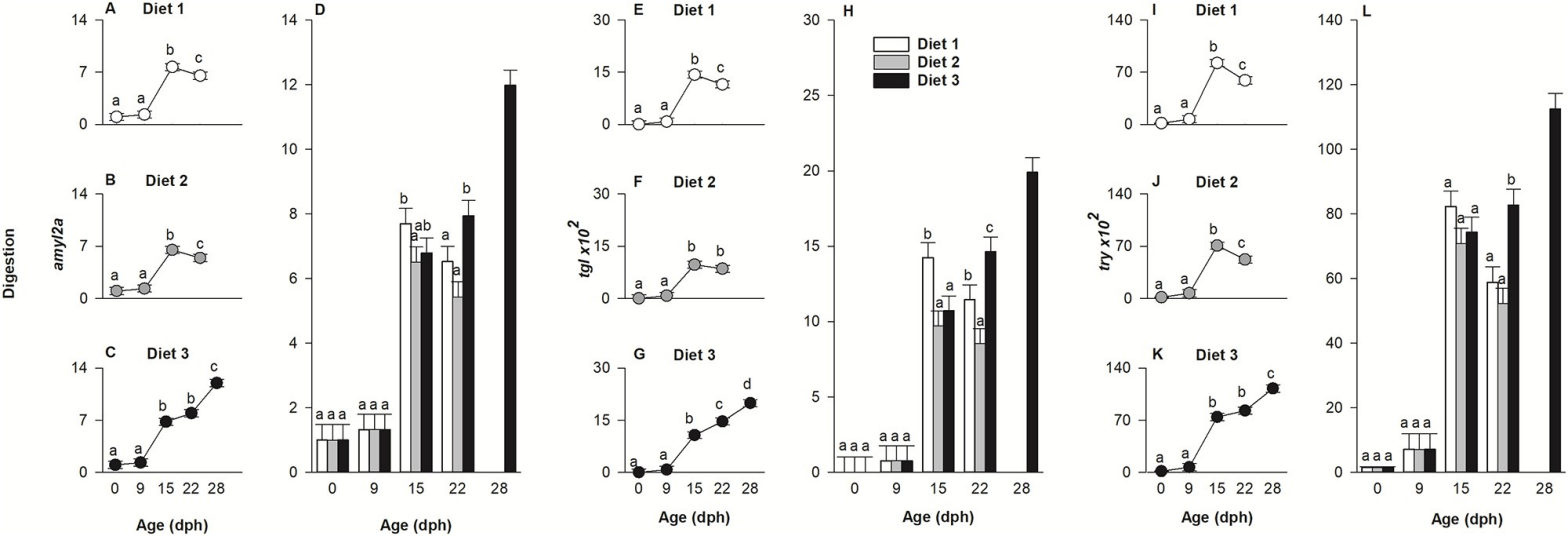
Relative expression of amylase (*amyl2a*), triglyceride lipase (*tgl*) and trypsin (*try*) in European eel, *Anguilla anguilla*, larvae. Effect of treatment (diet) at each age (A-C, E-G, I-K) and effect of age (days post hatch (dph)) at each treatment (D, H, L). Values represent means (± SEM), while different lower-case letters represent significant differences (p < 0.05).

In this study, the expression of genes related to digestion (*amyl2a*, *tgl*, *try*) were affected by the diet × age interaction (Fig 5; p < 0.01). Thus, the model was decomposed into a series of reduced one-way ANOVAs to determine the effect of age on gene expression for each diet and the effect of diet on gene expression at each age.

The expression of *amyl2a* significantly increased approximately 5-fold between hatch and 15 dph in the larvae, independently of the dietary treatment (p < 0.001). However, beyond 15 dph, the transcript level decreased for larvae fed diet 1 and 2 (Fig 5A and 5B; p < 0.01), while it continuously increased for larvae fed diet 3 (p < 0.0001), reaching peak values on 28 dph (Fig 5C). Moreover, the expression of *amyl2a* was higher for larvae fed diet 1 compared to diet 3 at 15 dph (p < 0.01), while at 22 dph it was highest for diet 3 fed larvae (Fig 5D; p < 0.01).

Similarly, the expression of *tgl* increased more than 1000-fold between 0 and 15 dph (p < 0.01). After this point, however, the expression of this gene decreased for larvae fed diet 1 (Fig 5E; p<0.001), while remaining stable for larvae fed diet 2 (Fig 5F), but it significantly increased for larvae fed diet 3, reaching peak values on 28 dph (Fig 5G; p < 0.01). As evident from Fig 5H, the expression of *tgl* was highest for larvae fed diet 1 at 15 dph (p < 0.01), but highest for larvae fed diet 3 at 22 dph (p < 0.01).

The expression of *try* followed a similar pattern as *amyl2a* and *tgl*, i.e. increasing approximately 50-fold towards 15 dph (p < 0.001). However, while increasing for larvae fed diet 3 (Fig 5K; p < 0.01), expression of *try* decreased for larvae fed diet 1 and 2 (Fig 5I and 5J; p < 0.001). Moreover, no significant difference was observed at 15 dph, while at 22 dph, the expression of *try* was higher in larvae fed diet 3 compared to the other experimental groups (Fig 5L; p < 0.01).

#### Genes related to appetite and feed intake

**Fig 6:**
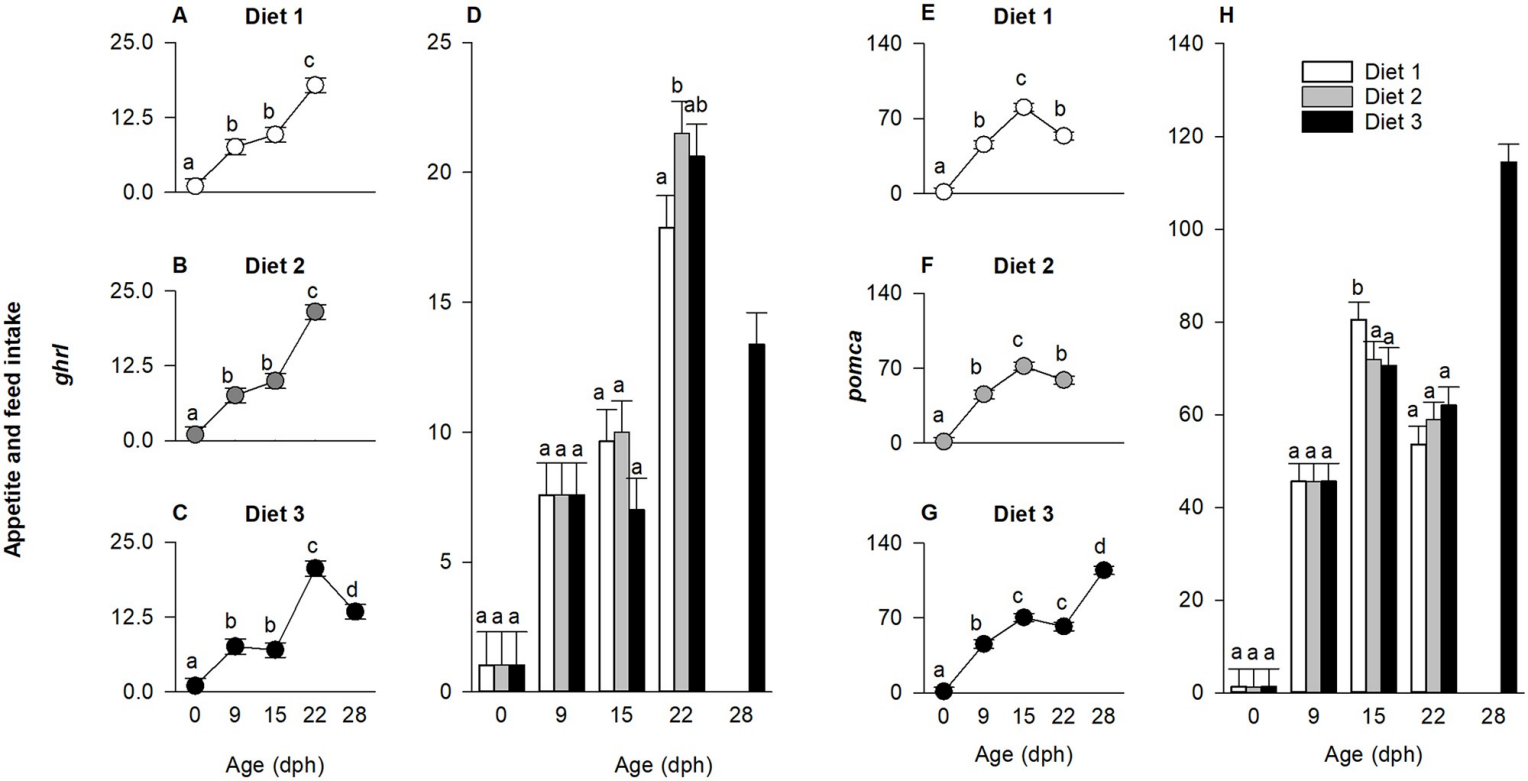
Effect of age (days post hatch (dph)) and treatment (diet) on relative expression of ghrelin (*ghlr*) and proopiomelanocortin (*pomca*) in European eel, *Anguilla anguilla*, larvae. Values represent means (± SEM), while different lower-case letters represent significant differences (p < 0.05).

The expression of genes regulating appetite and feed intake (*ghrl* and *pomca*) were affected by the diet × age interaction (p < 0.01). Thus, the model was decomposed into a series of reduced one-way ANOVAs (Fig 6D and 6H).

Expression of the gene encoding ghrelin (*ghrl*) increased 20-fold from hatch until 22 dph (Fig 6A–6C; p < 0.01), while it decreased beyond this point for larvae fed diet 3 (Fig 6C; p < 0.01). At 9 and 15 dph, *ghrl* expression did not differ among diets, however, at 22 dph, in larvae fed diet 1, *ghrl* expression was downregulated compared to diet 2 (Fig 6D; p < 0.05).

Independently of diet, the larval expression of *pomca* increased approximately 50-fold until 15 dph (Fig 6E–6G; p < 0.01). Beyond that point, *pomca* expression decreased for larvae fed diet 1 and 2 (Fig 6E and 6F; p < 0.01), while remaining constant between 15 and 22 dph for larvae fed diet 3. A significant upregulation with an 80-fold increased expression compared to the level at hatch was observed at 28 dph for larvae fed diet 3 (Fig 6G; p < 0.01). Moreover, at 15 dph the expression of *pomca* was highest in larvae fed diet 1, while no difference between treatments was observed at 22 dph (Fig 6H; p < 0.01).

#### Genes related to growth and energy metabolism

**Fig 7:**
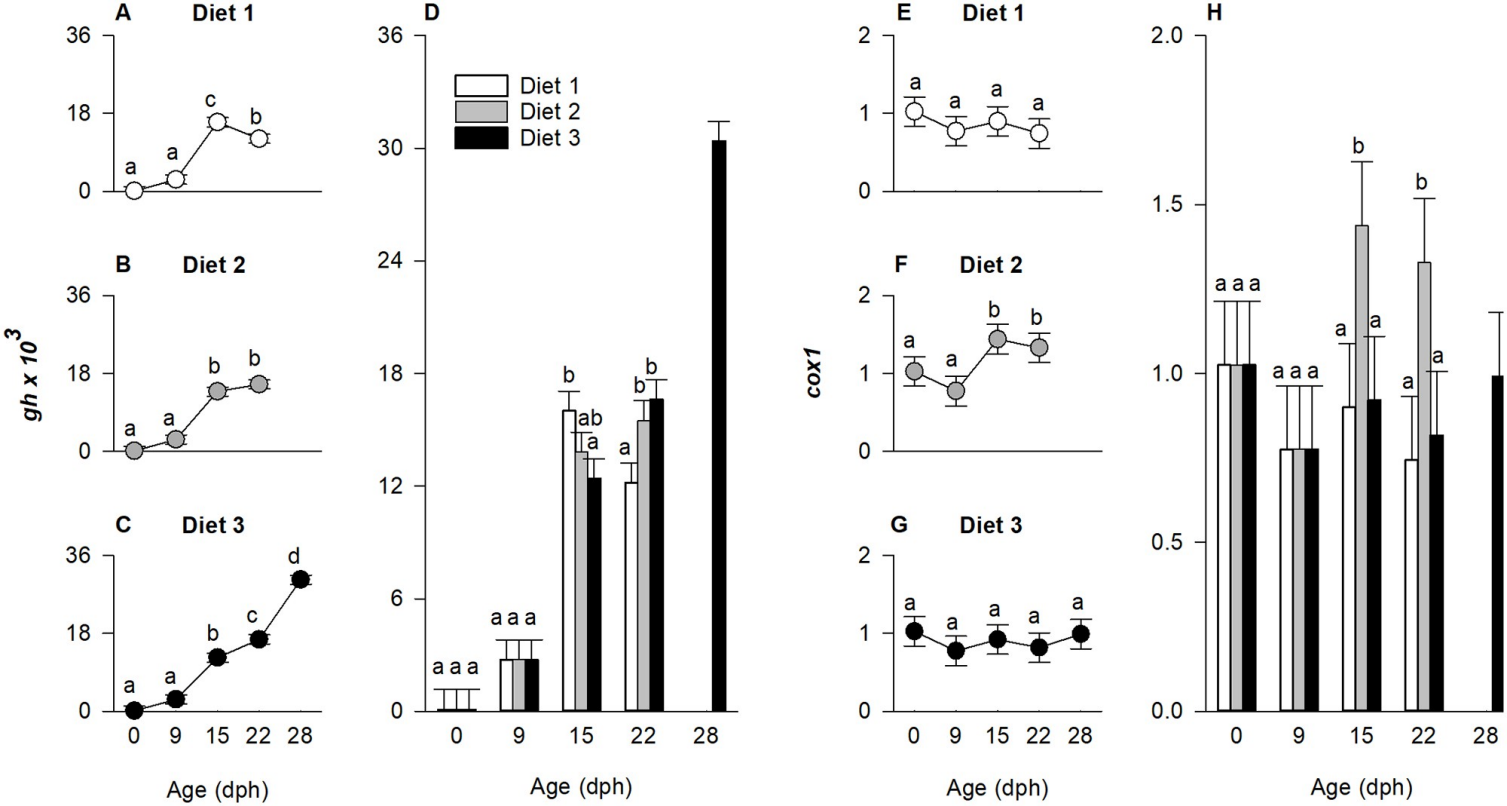
Effect of age (days post hatch (dph)) and treatment (diet) on relative expression of growth hormone (*gh*) and cytochrome c oxidase (*cox1*) in European eel, *Anguilla anguilla*, larvae. Values represent means (± SEM), while different lower-case letters represent significant differences (p < 0.05).

The expression of *gh* (growth) and *cox1* (energy metabolism) was significantly affected by the diet × age interaction (p < 0.01). Thus, the model was decomposed into a series of reduced one-way ANOVAs to determine the effect of age on gene expression for each diet (Fig 7A–7C and 7E–7G) and the effect of diet on gene expression at each age (Fig 7D and 7H).

Overall, *gh* expression increased until 15 dph (p < 0.01). Beyond 15 dph, the expression level decreased in larvae fed diet 1 (Fig 7A; p < 0.01), remained constant for diet 2 (Fig 7B), while significantly and progressively increased for larvae fed diet 3 (Fig 7C; p < 0.01). Moreover, the expression of *gh* was higher in larvae fed diet 1 compared to diet 3 at 15 dph (p < 0.01), while it was higher for larvae fed diet 2 and 3 compared to diet 1 at 22 dph (Fig 7D; p < 0.01). Markedly, in eel larvae fed diet 3, the expression of *gh* reached peak values on 28 dph, approximately 2-fold higher compared to 22 dph and around 300-fold upregulated compared to the level at hatch (Fig 7C and 7D).

The expression of *cox1* remained constant throughout the experimental period for larvae fed diet 1 and 3 (Fig 7E and 7G), while it was upregulated on 15 and 22 dph for larvae fed diet 2 (Fig 7F; p < 0.001). At 15 and 22 dph, the expression of *cox1* was higher in diet 2-fed larvae compared to the other experimental groups (Fig 7H; p < 0.01).

#### Standardized relative expression of selected genes

**Fig 8:**
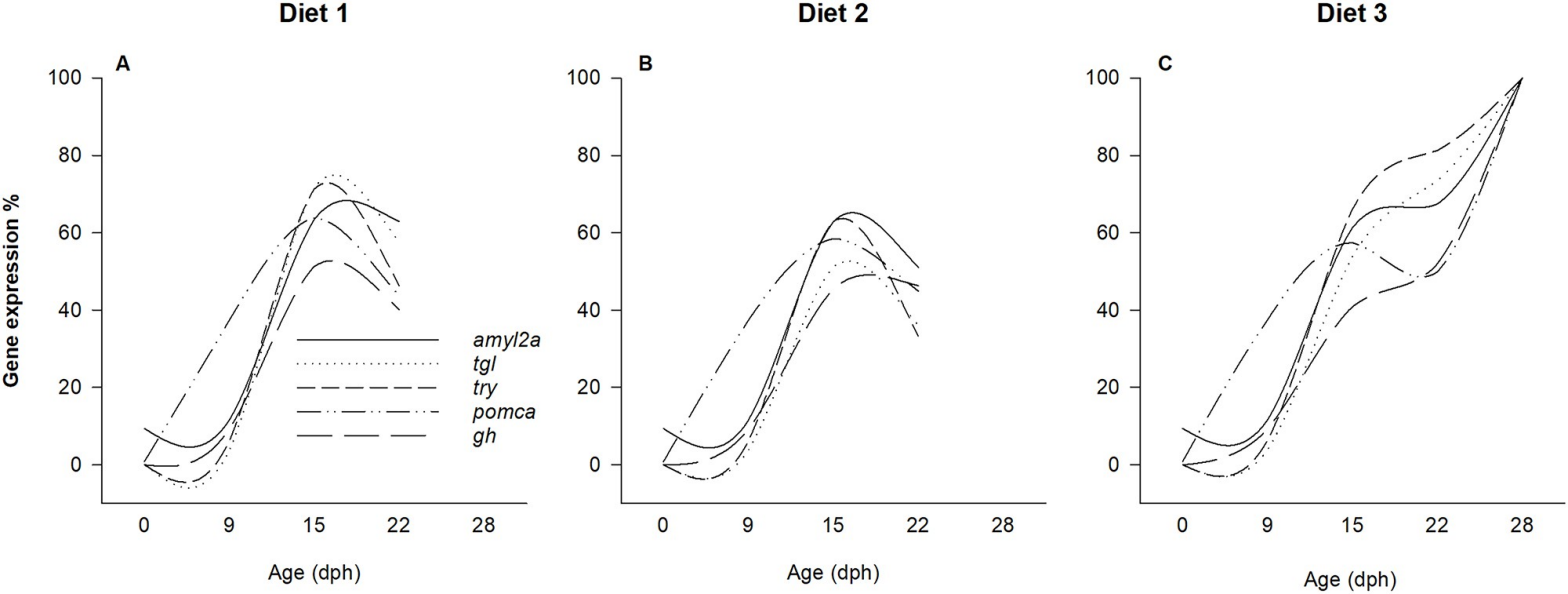
Relative expression of selected genes. Overview, showcasing the difference in expression of selected genes standardized to the highest recorded transcript of that gene (*try*, *amyl2a*, *tgl*, *pomca* and *gh*) in European eel, *Anguilla anguilla*, larvae fed diet 1 (A), diet 2 (B) and diet 3 (C) throughout the experimental period (from 0 to 28 days post hatch (dph)).

Among the analyzed genes, 5 out of 7 genes relating to digestion (*try*, *tgl*, *amyl2a*), food intake (*pomca*) and growth (*gh*) showed a steep increase in expression between 9 and 15 dph, however, after this time point, the pattern differed among treatments (diets). To visualize the diet specific expression pattern of those genes, the relative quantity of each target gene transcript (ΔΔCt value) was standardized to the highest recorded transcript of that gene in this experiment and expressed as a percentage (%) in Fig 8.

## Discussion

In the present study, three diets (diet 1, 2 and 3) were formulated and tested for establishing first feeding culture of European eel. Overall, eel larvae successfully ingested all diets tested, with an increasing feeding incidence and gut fullness from 15 to 22 dph. Interestingly, at 15 dph, larvae fed diet 1 were longer and transcripts of genes related to digestion (*tgl*) and feed intake (*pomca*) were upregulated (compared to larvae fed diet 2 and 3), which in combination with highest survival registered for larvae fed diet 1 on 17–20 dph, indicated a possible initial benefit of this diet for first-feeding European eel larvae. On the other hand, the generally higher gut-fullness combined with the improved survival, bigger size (length and body area), and higher dry weight from 22 dph and onwards of larvae fed diet 3, suggests that this diet had components benefitting eel larvae during this larval ontogenetic phase. Moreover, insights regarding the molecular ontogeny of first-feeding associated mechanisms and processes revealed that at 22 dph, genes related to digestion (*try*, *tgl*, and *amyl2a*), feed intake (*pomca*) and growth (*gh*) showed higher expression in larvae fed diet 3 compared to diet 1 and 2. In this regard, for larvae fed diet 1 and 2, expression of these genes peaked at day 15, but showed a “dropping” pattern towards 22 dph, indicating that these diets lead to a decline in molecular functionality of those aforementioned processes. In contrast, for larvae fed diet 3, the expression patterns of *try*, *tgl*, *amyl2a*, *pomca*, and *gh* continued to increase towards 28 dph, indicating successful exogenous feeding initiation and an improved maturation and functionality of the corresponding molecular mechanisms relating to feeding, digestion, and growth.

Overall, this study reports the so far highest registered survival of hatchery cultured European eel larvae, where 4% of larvae fed diet 3 survived until the end of this experiment (28 dph), compared to 0.5% of larvae fed diet 2, while those fed diet 1 did not survive until that stage. In nature, mortality during early life stages of fish is expected to be high with estimated averages of 96.40% and 99.98% for freshwater and marine species, respectively [40]. From an aquaculture perspective, 4% survival is low, however, when the development of Japanese eel larval culture technology was at a comparable stage, Japanese eel larvae showed survival rates similar to the ones reported in this study, ranging between 3 to 5% [18]. Interestingly, in the present study, the larvae fed the Japanese based diet 1 did not survive long beyond the point of no return. This might indicate that either the formulation for this diet differs from the Japanese original or that larval nutritional requirements slightly differ between species, but possibly also that current feeding processes and techniques need to be improved. Nevertheless, the survival rate observed in the present study, reaching 51.9% at 20 dph, is substantially higher than the previously reported survival rate (21.6%) for European eel larvae at the corresponding age [22]. However, further research is still needed to improve diet formulations and dietary regimes in order to enhance growth and survival rate in European eel larval culture.

Regarding feed formulation, the amount of lipids included in a diet directly affects larval performance. Thus, decreasing dietary lipid content (from 30 to 10%) can enhance digestive tract maturation and improve larval development in European seabass, *Dicentrarchus labrax* [41]. Similarly, for Japanese eel larvae, it was observed that the utilization of a diet based on defatted shark eggs (where the lipid content decreased from 40.4% to 26.3%) led to an increased larval survival and growth performance [34]. Overall, there is increasing evidence that the appropriate amount of lipids in marine fish larval diets should be lower than 30% [42]. As such, the reduction in lipid concentration applied in the present study (i.e. from 40% in diet 1 to 30% in diet 3) seems to be a step in the right direction, but needs to be further addressed in future studies.

Together with lipids, also aspects regarding quality and quantity of dietary proteins are directly affecting larval development and survival [43]. Due to the high growth potential of fish larvae, the type of dietary proteins and their digestibility are key factors for their healthy development and survival [44, 45]. In this study, in addition to the main protein fraction originating from dogfish egg yolk, diet 2 and 3 included extra sources of proteins such as hydrolysates and whey, respectively. In this regard, proteins from hydrolysates (commonly di- and tripeptides with molecular weights of ~3 kDa), are efficiently absorbed by the intestine and do not need to be pre-digested by the pancreatic enzymes [46, 47]. Therefore, including hydrolyzed proteins in fish larval diets has been demonstrated to enhance survival and development of digestive functionality as well as resistance to pathogens in European seabass, *Dicentratus labrax* [48, 49], survival of goldfish, *Carassius auratus* larvae [50] and growth of spotted wolfish, *Anarhichas minor* larvae [51]. Consequently, incorporation of protein hydrolysates in diet 2 would be expected to improve growth and survival of eel larvae. However, eel larvae fed diet 2 showed lower survival and growth compared to larvae fed diet 3. In this regard, inclusion of too high proportions of hydrolyzed protein appears to have had negative effects on larval performance, which similarly was reported for Senegalese sole, *Solea senegalensis* [46], gilthead seabream, *Sparus aurata* [52, 53], Asian sea bass, *Lates calcarife* [54] and Atlantic halibut, *Hippoglossus hippoglossus* [55, 56]. In those cases, the lower larval performance was attributed to a saturation of the transport system in the intestinal brush-border membrane due to overloading of short peptides (di- or tripeptides), causing an imbalanced utilization of free amino acids and resulting in a decreased protein accretion [57]. On the other hand, the inclusion of whey (molecular weight of approximately 10 kDa) as an extra source of protein in diet 3, seems to have benefitted the European eel larvae over time, especially beyond 22 dph. These results point towards the benefit of inclusion of more complex dietary protein (as in diet 3) for eel development and survival throughout and beyond the first feeding window.

At this point, it is worth mentioning that two periods of high mortality were identified in the present study. The first period occurred shortly after the first introduction of feeds at 10 dph, which may be driven by the challenging transition from endogenous to exogenous feeding processes, where larvae undergo critical developmental changes in organogenesis and ontogeny. At the same time, the current larval culture protocol implemented changes in rearing conditions for first-feeding eel larvae, including new tank hydrodynamics and design (moving from a 77 L tank to a 8 L Kreisel tank), a reduction in salinity as well as changes in water mass (change in water microbiota when moving to new RAS units). Applying these changes in rearing techniques, contribute to the optimized protocols for accommodating feeding procedures. However, maintaining water quality within the rearing tanks becomes a challenge, especially in connection with initiating larval feeding on such liquid diets, as it requires a lot of manual handling and daily changes of the tanks to prevent negative microbial interference in connection to the contamination of the tank walls [20]. Therefore, besides enhancing the quality of diets, also other aspects for development of future hatchery production, such as the impact of feeding inert diets on water quality, tank design and hydrodynamics as well as smart larval transfer solutions, need attention.

The second period of high mortality occurred around 20–24 dph despite that feeds were ingested. Such mortalities are common for fish larvae that do not successfully take up first-feeding, initiate feeding too late or receive an unsuited diet. Despite the presence of feed items, the ability to search, capture, and/or assimilate nutrients may fail, leading to irreversible mortality [58, 59]. Thus, in the present study, the eel larvae may have reached the “*point of no return*”. This can be supported by the continuous upregulation of *ghrl*, which irrespectively of the diet, peaked at 22 dph, indicating that most eel larvae were hungry and potentially fasting, as this gene encodes the peptide ghrelin, which is referred to as the “*hunger hormone*”. In contrast, larvae fed diet 3, downregulated the expression of *ghrl* beyond 22 dph, which could indicate that those larvae established first-feeding and thus, were not starving, resulting in survival beyond the “*point of no return*”. As such, this study documents for the first time the ontogeny of feeding European eel larvae surviving beyond the point of no return with signs of growth. However, while Diet 3 sustained survival at a low level, further research regarding dietary requirements will be needed in order to increase survival.

## Conclusions

Overall, a potential initial benefit of diet 1 was justified by higher survival within the first-feeding window as well as bigger size and higher expression of genes related to digestion (*tgl*) and food intake (*pomca*). However, the inclusion of more complex dietary proteins as in diet 3, but not hydrolyzed peptides as in diet 2, promoted larval ontogeny after the successful transition to exogenous feeding. This led to higher survival of diet 3 fed larvae beyond the first-feeding stage with morphological and molecular advantages compared to larvae fed diet 1 and 2. Together, the results of the present study suggest that the digestive and assimilation capacity of European eel larvae may vary throughout ontogeny and thus, the dietary regime may need to be adapted to meet developmental stage-specific requirements and/or preferences. Overall, the present study provides an unprecedented step towards establishing diets and first-feeding culture of hatchery-produced European eel larvae as basis for future sustainable aquaculture of this high-value species.
